# Analysis of the Relationship between the Psychological Well-Being, Emotional Intelligence, Willpower, and Job-Efficacy of Clinical Nurses: A Structural Model Application

**DOI:** 10.3390/ijerph18115582

**Published:** 2021-05-24

**Authors:** Jin-Hwa Lee, In-Ok Sim

**Affiliations:** Department of Nursing, Red Cross College of Nursing, Chung-Ang University, 84 Heukseok-ro, Dongjak-Gu, Seoul 06974, Korea; loveljh5@naver.com

**Keywords:** clinical nurse, job-efficacy, psychological well-being, emotional intelligence, willpower

## Abstract

The aim of this study to discover the relationship between psychological well-being, emotional intelligence, willpower, and job-efficacy. The data were collected from 26 May to 30 May 2020 by distributing a questionnaire to 317 clinical nurses with six months of experience in a general hospital located in Seoul. Three hundred copies were collected and used for final data analysis. The results of the study verified that the direct factors of psychological well-being, emotional intelligence, and willpower affect the job-efficacy of clinical nurses and confirmed that emotional intelligence is a mediating factor between psychological well-being and job-efficacy. This study is meaningful in that it proves the necessity of establishing various curriculums focusing on these factors so that nursing students can best perform their duties as professional nurses. In particular, it is suggested that an educational program and curriculum be established that can strengthen the psychological well-being and enhance the emotional intelligence of nursing students. It is expected that such training will equip professional clinical nurses to effectively handle future work in their stress-filled field.

## 1. Introduction

As access to medical information becomes easier, consumers are growing in their familiarity with medical institutions, and the level of expectation for specialized services is increasing [1,2]. To enhance competitiveness, hospitals provide high-level medical services to improve patient satisfaction [3]. Nurses constitute the largest number of personnel in a hospital organization. They are closest in proximity to the patients with the services they provide and are an important human resource in establishing strategies for improving medical services. Satisfaction with the nursing services provided greatly influences a patient’s intention to reuse the hospital [4]. Medical institutions are very interested in cultivating excellent nursing manpower to increase the competitiveness of their hospitals and are preparing plans to retain them in the organization [5].

Recently, many studies have focused on job-efficacy in relation to the burnout, emotional labor, and organizational performance demonstrated by nurses in their work [6,7]. Job-efficacy has important implications for a job [8] and is a measure that enables individuals to judge their ability to organize and perform a given task [9]. In the nursing field, job-efficacy is an important concept that affects job performance. It can be a driving force in motivating individual nursing activities and stimulating their research activities [10]. 

Kang and Ko (2006) reported that the higher the job-efficacy of a nurse, the more positively it affects the whole profession [11]. When job-efficacy is high, it improves the effectiveness of individuals and organizations by providing high-quality nursing services to patients. It is also said that job-efficacy helps relieve stress on individuals and organizations. Furthermore, nurses with high job-efficacy typically have positive outlooks on the future and increased satisfaction with their quality of life. Thus, it is necessary to search for factors related to job-efficacy [12].

Nurses experience tension in various clinical situations they encounter while caring for patients. They often face difficulties in controlling their emotions due to lack of practical medical autonomy to cope with medical needs [13]. Nurses should always be positive and friendly when dealing with patients. However, the self-control this involves can sometimes be a burden, as nurses have to hide and suppress negative emotions, such as anger and disappointment [14]. 

In addition, nurses play an intervening role by providing appropriate treatment through monitoring and early detection to save a patient’s life [15]. Due to these roles, nursing is a field rife with tension, and nurses can experience uncertainty about their identity in the clinical field.

For nursing specialists, the continuous emotional nature of their work increases burnout and workplace stress, and nurses can become cynical in their relationship with patients or co-workers, resulting in reduced efficiency at work [16,17]. Halbesleben, Rathert, and Williams reported that nurses experience significantly higher emotional burnout than those in other occupations, which is a factor that lowers job-efficacy and job satisfaction [17]. Nurse burnout is common and can have a very negative impact on a patient’s medical treatment.

Emotional and psychological anxiety, especially, negatively affects the running of a hospital by causing problems such as a decline in nurses’ efficacy in performing their duties, a decrease of the trust level between patients and nurses, and a greater turnover of nurses [18]. For the efficient manpower management of medical institutions, as well as of individual nurses, hospitals should take care to maintain psychological well-being and reduce the emotional problems related to emotional burnout, which will, in turn, enhance the job-efficacy of nurses.

Recent research describes the positive effects of emotional intelligence on job-efficacy and psychological well-being [19]. Emotional intelligence is the positive psychological tendency to accurately understand the emotions of oneself and others in various situations and to control and utilize one’s emotions according to the situation [20]. It is one of the psychological resources available to individuals. Since nurses are professionals who provide people-centered assistance, high emotional intelligence is required. This is because nurses must express their feelings with appropriate behavior according to each medical situation they encounter, and even if they face a challenging situation, they must be able to evaluate it positively and cope with it in a desirable manner [21]. People with low emotional intelligence cause emotional conflict and have a high level of stress perception, making it difficult for them to adapt to social situations. People with high emotional intelligence, however, are known to evaluate positively and exhibit desirable coping behavior even under stressful situations [22]. Therefore, emotional intelligence can mediate job stress, burnout, and job turnover for nurses working in a severely emotional field [23]. In addition, emotional intelligence is a very important factor in coping with emotional work and improving psychological well-being through effective emotional treatment [24], as well as job-efficacy. Therefore, it is necessary to conduct a comprehensive study of the relationship between them so that the emotional health and working environment of nurses can be ameliorated.

In recent studies on clinical nurses, interest in the relationship not only between psychological well-being and emotional intelligence, but also between psychological well-being and willpower, has increased [22,25]. Psychologists have argued that willpower is an important factor they consistently discover when identifying personality traits that lead to a more positive future [26]. Willpower refers to self-control, a force that overcomes temptation. Thoughts on this are divided into two theories: the limited resource theory, which states that willpower is a limited resource that is exhausted after difficult mental activities, and the nonlimited resource theory, which asserts that it is possible to continue a difficult mental activity indefinitely [27]. The individual belief in willpower is related to psychological well-being. However, belief in the nonlimited theory has a positive effect on psychological well-being, whereas belief in the limited theory has a negative effect [26]. It is reported that controlling oneself to consistently exert willpower has a positive relationship on psychological well-being [28,29]. A job characteristic of nurses is to perform treatment based on information collected about a given patient and procedure within a limited time. Thus, willpower is needed to control or delay the immediate desire to achieve goals in order to balance personal health, life, and nursing work. This study aimed to verify the structural path that forms the relationship between psychological well-being, emotional intelligence, willpower, and job-efficacy. In addition, the intent was to provide basic data that can help improve the quality of life to preserve the psychological well-being and effective work performance of nurses and so maintain professionalism in nursing care.

## 2. Materials and Methods

### 2.1. Aims

The aim of this study was to discover the relationship between psychological well-being, emotional intelligence, willpower, and job-efficacy. In this study, the research hypotheses to solve the research problem were set as follows:

1. The psychological well-being of nurses has a positive effect on emotional intelligence.
2. The psychological well-being of nurses has a positive effect on willpower.
3. The psychological well-being of nurses has a positive effect on job-efficacy.
4. The emotional intelligence of the nurse has a positive effect on job-efficacy.
5. The nurse’s willpower has a static effect on job-efficacy.

### 2.2. Design 

**Figure 1:**
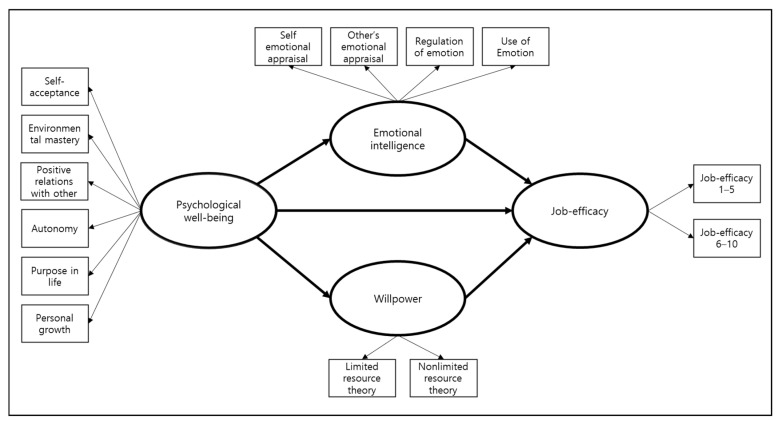
Study model.

This study is a path analysis study to verify the suitability of the hypothesized relationships by establishing a hypothetical model for the psychological well-being, emotional intelligence, will power, and job efficacy of clinical nurses as presented in Figure 1.

### 2.3. Participants

Participants in this study surveyed subjects with more than 6 months of experience working as a nurse at S City General Hospital. In order to use the structural equation model, more than 200 sample sizes suitable for the maximum likelihood method were described, so 317 subjects who agreed to participate according to this sample criterion were sufficiently recruited and the questionnaire was conducted [30]. However, in the coding process, questionnaires of subjects with insufficient data answers were excluded, and finally 300 nurses participated as the subjects of this study.

### 2.4. Research Instruments

The survey was conducted through a self-report questionnaire. The contents and research purpose of data management and the confidentiality plan were explained to the participants. Additionally, the questionnaire was conducted for those who had signed the research participation manual and research participation agreement. The survey was conducted from 26 May to 30 May 2020. The purpose and method of the study were explained by visiting the hospital nursing department, which was randomly selected, and the questionnaire was distributed with permission to collect data.

#### 2.4.1. Psychological Well-Being

Psychological well-being is explained as the sum of psychological aspects that are thought to affect the quality of life of an individual [31]. In this study, the psychological well-being scale (PWBS) developed by Ryff [31], and adapted to Korean culture by Kim, Kim, and Cha, was used [32]. Sub-concepts that constitute psychological well-being include self-acceptance, positive relations with others, autonomy, purpose in life, and personal growth. This scale is a 46-item version, and is a Likert-type 5-point scale that ranges from 1 (strongly disagree) to 5 (strongly agree). The total score ranged from 46 to 230, and the higher the score, the higher the psychological well-being. The reliability of this scale was Cronbach’s α = 0.91. In this study of Cronbach’s α = 0.91 was measured.

#### 2.4.2. Emotional Intelligence

Emotional intelligence is a positive personal emotional orientation that is the ability to use and control one’s emotions while accurately understanding oneself and the feelings of others [20]. In this study, the Korean emotional intelligence scale adapted by Kim and Yoo was used [33]. This scale consists of four sub-factors. There are 16 questions, including “Self-Emotional Appraisal,” “Other’s Emotional Appraisal,” “Regulation of Emotion,” and “Use of Emotion.” It was originally developed by Wong and Law [20]. In the study by Kim and Yoo, Cronbach’s α = 0.79 was measured. The scale was Cronbach’s α = 0.90 in this study.

#### 2.4.3. Willpower

Willpower is the ability to control oneself and restrain impulses. Beliefs about this mental strength are divided between the limited resource theory, the belief that willpower is a limited resource, and the nonlimited resource theory, the position that willpower is unlimited. This study used the “Implicit Theory of Willpower for Strenuous Mental Activities Scale” that Ha and Cho adapted and validated for Korea [34]. This scale consists of a total of six questions, with three questions each on willpower’s limited and unlimited nature. It is a Likert-type 5-point scale from 1 (Strongly disagree) to 5 (Strongly agree). This scale was a self-reported questionnaire. In the study of Ha and Cho [34], Cronbach’s α = 0.93 was measured. Cronbach’s α was 0.84 in this study.

#### 2.4.4. Job-Efficacy

Job-efficacy refers to the belief that something can be done and is a human’s internal predisposition [35]. The job-efficacy scale used on the self-reported questionnaire was the Personal Efficacy Beliefs scale developed by Riggs and Knight [36]. This refers to the score measured by using the scale of 10 questions adapted by Kang and Ko [11]. It was a Likert-type 5-point scale from 1 (Strongly disagree) to 5 (Strongly agree), and negative items were reverse-scored. Cronbach’s α was 0.73 in Kang and Ko [11]. In this study, Cronbach’s α = 0.83 was measured.

### 2.5. Ethical Considerations

This study was approved by the Institutional Review Board of C University in Seoul (IRB No.1041078-202003-HRSB-065-01) for the protection of participants, and data were collected with the permission of the hospital. The purpose of this study was explained through consent forms given to the participants, and only those who understood the purpose and procedure of this study and agreed to the questionnaire took part. It was explained to the participants that they could terminate their participation at any time according to their wishes. The consent form contains information on the participant’s anonymity and confidentiality, and the research data will be discarded three years after the research paper is published in print, in accordance with the hospital ethics regulations.

### 2.6. Data Analysis

This study processed computational statistics using SPSS 21.0 (IBM, Armonk, NY, USA) and AMOS 22.0 (IBM, Armonk, NY, USA) programs to analyze the relationship between psychological well-being, emotional intelligence, willpower, and work efficacy according to the research purpose. A structural equation modeling was used to examine a hypothesized model, and the data were analyzed in the following way:

1. A frequency analysis was conducted to understand the demographic characteristics of the study participants.
2. To confirm the normality of the measured variable, the skewness and kurtosis of each variable were analyzed and confirmed.
3. The correlation and multicollinearity between the measured variables were analyzed by the Pearson correlation coefficient.
4. Confirmatory factor analysis (CFA) was conducted to confirm the fit of the measurement model and the reliability and validity of the latent variable.
5. A measurement model analysis was performed to confirm whether the variables representing the latent variables of this study, psychological well-being, emotional intelligence, willpower, and job-efficacy, adequately represented the latent variables.
6. Structural equation model analysis was conducted to verify the fitness of the established research model. This was determined by passing the determined criteria indicators. The model fit indices used were the goodness of fit index (GFI), the adjusted goodness of fit index (AGFI), the root mean square residue (RMSR), the normal fitness index (NFI), the relative fitness index (RFI), the increased fitness index (IFI), the Tucker-Rui index (TLI), and the comparative fitness index (CFI).
7. In the structural equation model, the bootstrapping approach was used to verify the significance of direct effects, indirect effects, and total effects among variables. Using the bootstrapping method provided by the AMOS 22.0 statistical program, we analyzed the mediating effect set in the research hypotheses.

## 3. Results

### 3.1. Demographic Characteristics of Participants

The study participants ranged in age from 31 to 40 years. The general characteristics of the participants in this study through descriptive statistics are shown in Table 1.

### 3.2. Descriptive Statistics and Correlations

Table 2 shows the results of average and standard deviation related to the sub-factors of psychological well-being, emotional intelligence, willpower, and job-efficacy, which were the main variables of this study. The psychological well-being score was 2.945 ± 0.35 (scale range: 1–5), the emotional intelligence measured 3.56 ± 0.61 (scale range: 1–5), the willpower score was 3.6 ± 0.68 (scale range: 1–5), and the job-efficacy measured 3.60 ± 0.61 (scale range: 1–5).

Pearson’s product–moment correlation analysis was used to investigate the correlation between the observed variables. The results of the correlation analysis are shown in Table 3.

Psychological well-being, emotional intelligence, job-efficacy, and willpower all showed positive (+) significant correlations with each of the sub-factors. Pearson’s correlation coefficient was less than 0.8, indicating that there was no multicollinearity problem.

### 3.3. Measurement Model Analysis

#### 3.3.1. Fit of Measurement Model

The goodness of fit index of the Confirmation Factor Analysis (CFA) model was used to determine how well the measurement model used in the study fit the sample data. The fitness index selected in this study used the absolute fitness index, the incremental fitness index, the simple fitness index and other indices (11 types), which are presented as the minimum fitness index, and the results are shown in Table 4. All goodness-of-fit indices met the standard. The chi-square test was conducted to evaluate the model. The chi-square test gave a value of = 226.87; df = 70; *p* < *0*.001. As a result, this model reflects the actual data, which was consistent with the sample data and was recognized as reliable.

#### 3.3.2. Validity of the Measurement Model 

In the measurement of the structural equation model, validity is verified by measuring one latent variable as multiple observed variables. To confirm whether the observed variable could reasonably explain the latent variable, a factor loading analysis was conducted indicating concentration validity. Structural equation path coefficients represent standardized regression weight, standard error, and critical ratio (C.R.). As a method of verifying the intensive validity, it is judged suitable if the factor load of the variable is 0.4 or more. The results of path coefficient analysis based on the above criteria are shown in Table 5.

In this study, since the C.R.s of the measurement model were all 6.22 or higher than the standard 1.96, it was found to be significant at the significance level of 0.01. Since there was no subscale for job-efficacy, the number of individual questions was large, and as a result, the number of unknowns that must be estimated increased substantially. Therefore, the scale was divided into two with item parceling methods and analyzed. For all observed variables, every factor loading was more than 0.4. Therefore, the convergent validity of the measurement model was verified. Accordingly, a structural equation model analysis was performed without adding or removing observed variables.

### 3.4. Structural Model Verification

**Figure 2:**
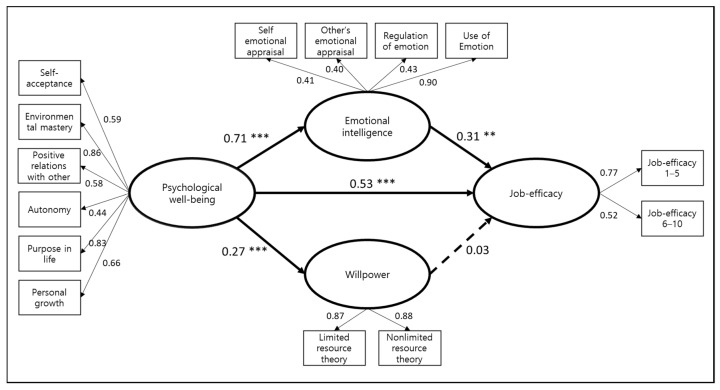
Final model. Note: ** *p* < 0.01, *** *p* < 0.001.

Through the measurement model analysis, it was confirmed that the latent variable was properly measured for the observed variable. Next, a structural equation model analysis was conducted. Using AMOS 22.0 statistical program, the parameters of the structural model set in the research model were estimated. The maximum likelihood (ML) was used as a parameter estimation method. The analysis results and the final model of the study are shown in Figure 2. All figures and tables should be cited in the main text as Figure 1, Table 1, etc.

#### 3.4.1. Relationships between the Human Effects of the Measurement Model

This study explored whether there was a direct relationship between the psychological well-being, emotional intelligence, willpower, and job-efficacy of the participating nurses. The result of analyzing the path coefficient of the final model is shown in Table 6.

The standardized regression weight (SRW) between the nurses’ psychological well-being and all other variables was found to have a statistically significant positive effect. 

In the relationship between emotional intelligence and job-efficacy, the standardization coefficient was found to have a statistically significant positive effect. However, in the relationship between willpower and job-efficacy, the standardization coefficient was found to have no statistically significant effect. Therefore, hypothesis 5 was rejected in this study.

In Figure 2, the influence relationship between each variable of the final research model is expressed by considering the standardization coefficient and C.R.

#### 3.4.2. Verification of Direct and Indirect Effects

Based on the final research model, direct, indirect, and total effects were calculated by effect decomposition to verify the significance of mediating effects according to the hypotheses. Using the bootstrapping method provided by the AMOS 22.0 statistical program, we analyzed the mediating effect set in the research hypotheses. These are shown in Table 7. The indirect effect of psychological well-being on the path to job-efficacy through emotional intelligence was measured to be statistically significant at 0.229. Emotional intelligence can be considered to have a positive effect as a mediator between psychological stability and job-efficacy. However, it was determined that willpower had no indirect effect between the two variables in a mediating role in the relationship between psychological well-being and job-efficacy. As a result, it was ascertained that since psychological well-being has a statistically significant direct effect on job-efficacy, psychological well-being has a partial mediating effect that affects job-efficacy through emotional intelligence.

## 4. Discussion

The level of job-efficacy of a nurse as a professional medical practitioner greatly affects not only their own health and quality of life, but also the quality of nursing care provided for patients and the performance of the hospital. Recently, various research results have emphasized the importance of individual job-efficacy to maximize organizational performance. In particular, job performance ability affects hospital competitiveness [37].

The results of this study confirmed the importance of job-efficacy and identified a structural path for the factors that influence it. Accordingly, related matters will be discussed based on the research hypotheses.

### 4.1. The Relationship between a Nurse’s Psychological Well-Being, Emotional Intelligence, and Job-Efficacy

The psychological well-being of nurses incorporates mental and physical health, as well as mental well-being, and is a very important factor in the process of providing nursing care. Vermaak, Görgens-Ekermans, and Nieuwenhuize (2018) explained the relationship between psychological well-being and emotional intelligence, which manipulates stress to increase job-efficacy for nurses [38]. Yang reported that the psychological well-being and emotional labor of a nurse are related to job-efficacy, and higher psychological well-being leads to an increase of pleasant emotions and a decrease of unpleasant ones, which enhances job-efficacy [39]. In this study, the psychological well-being of nurses had an effect on the path to job-efficacy via emotional intelligence. Emotional intelligence had a positive effect as a mediator between psychological well-being and job-efficacy.

Emotional intelligence refers to the appropriate expression of emotions when complex emotions arise inside oneself. This theory, first used by Salovey, Mayer, Goldman, Turvey, and Palfai [40], pertains to an individual’s ability to better understand and control their own emotional state and that of others. In addition, it includes the ability to help solve organizational problems effectively by not responding to emotions immediately, but by acting in an appropriate manner in a given situation [41]. This study also confirmed the relationship between emotional intelligence and job-efficacy, including the recognition of one’s own emotions, the perception of others, use of emotions, and emotional regulation. These results suggest that emotional intelligence plays a mediating role between psychological well-being and job-efficacy and has a static effect. As reported by Lee and Song [41], the above results are consistent with the theory that emotional intelligence has a significant positive correlation with self-efficacy.

Emotional intelligence is gaining interest for its ability to promote the performance of high-level nursing by integrating nurses’ personal skills and patient-personal skills [42,43]. The results of this study are consistent with the studies of Pérez-Fuentes et al. (2019) that the higher the emotional intelligence level, the lower the job stress and the higher the job-efficacy [44]. Lee and Seo (2015) argued that job-efficacy has an effect on areas such as positive outcome expectation and job satisfaction, and this helps to develop into a sense of satisfaction with overall life [45]. Job-efficacy is the belief that someone can successfully perform the actions necessary to achieve the intended results. Even embracing this belief alone, people will approach their job more positively and have positive emotions for the whole of their lives. Higher job-efficacy leads to better stress control, higher self-esteem, and consequently more positive life satisfaction [46,47]. In this study, it was also confirmed that psychological well-being has a positively significant correlation with job-efficacy. This is consistent with the results presented in previous studies.

It can be seen, then, that psychological well-being, emotional intelligence, and job-efficacy are significantly related, and job-efficacy is an important concept not only to improve nurses’ ability to perform their duties, but also to improve their health behavior. Therefore, it is necessary to develop and apply educational programs that can improve psychological well-being and emotional intelligence to increase job-efficacy.

### 4.2. The Relationship between Clinical Nurses’ Psychological Well-Being, Willpower and Job-Efficacy

After examining the causal relationship between the nurses’ psychological well-being, willpower, and job-efficacy, the path between psychological well-being and willpower was found to be statistically significant. However, willpower had no significant indirect effects on the two variables of psychological well-being and job-efficacy. When dealing with personal qualities that predict positive outcomes in life, psychologists have commonly described limited and unlimited beliefs about willpower and explained that improving willpower is the surest way for a better life [27].

In addition, many studies on willpower also explain that people with unlimited beliefs tend to experience high subjective well-being and to be good at psychological regulation. It has also been reported that unlimited beliefs are positively correlated with self-regulation, psychological well-being, and academic achievement in college students [48]. In this study, willpower was shown to have a low influence on job-efficacy, so it was found that it did not support existing research results. The tools used in previous studies were the results of measurements for the general public and college students. However, it seems that the tools used in the past did not sufficiently reflect the professional characteristics of nurses, who require a high level of control for their tension-filled work. Therefore, though the results of previous studies so far have reported that there is a positive influence between willpower and job-efficacy, it is necessary to develop a valid research tool to research nurses, and further studies are needed to clarify the relationship between these two variables.

### 4.3. Limitations

This study shows that the direct effect of psychological well-being on job-efficacy is statistically significant, and psychological well-being has an effect on job-efficacy via emotional intelligence. Based on the results of this study, the following approaches are proposed for future studies: First, since this study is limited to the generalization of nurses in general hospitals in Seoul, repeated studies are recommended to consider various levels of each region and medical institution. Second, the results of this study showed that willpower did not play an important role as a mediating effect of psychological well-being and job-efficacy. However, other research results have reported a positive mediating effect. Therefore, this should be reviewed through subsequent studies. Third, it is necessary to develop a systematic theory based on the results of analyzing the causal relationship between psychological well-being and job-efficacy in this study. In the future, additional research could explore explanatory variables that affect job-efficacy.

Finally, further studies are suggested to develop a willpower measurement tool that considers the professional characteristics of nurses and to repeat research using this. In this study, a study was conducted on 300 general hospital nurses limited to similar areas of clinical nurses and hospital environments. Although the study was conducted by collecting samples that can describe the population, there may be limitations in that the same results may be reflected depending on the clinical nurse, hospital setting, and various characteristics of the area. Therefore, in the future, extensive and repetitive research on the same concept is needed in consideration of regional characteristics, medical institution level, hospital environment, and clinical nurses.

## 5. Conclusions

The purpose of this study was to establish a hypothetical model to verify the relationship between nurses’ psychological well-being, emotional intelligence, willpower, and job-efficacy. In the final model, an appropriate level of fitness was measured, excluding the relationship between willpower and job-efficacy.

The results showed that psychological well-being influences job-efficacy through emotional intelligence. Since emotional intelligence serves as a mediator between psychological well-being and job-efficacy, it is important to reinforce the factors of emotional intelligence. Additionally, psychological well-being is a factor that directly affects job-efficacy. It shows that job-efficacy can be improved only when it is preceded by the ability to control individual emotions efficiently while performing specific tasks. However, the result that willpower has little effect on job-efficacy suggests the following points: For nurses working in rapidly changing medical fields, such as emergency and trauma care, it means that considerable energy is needed to control tension and stress in order to increase willpower levels. There is a possibility, therefore, that other factors may act as mediators between willpower and job-efficacy, and that further studies are needed.

In conclusion, since this study shows that the relationship between psychological well-being, emotional intelligence, and job efficacy has a positive effect on nursing practice, concrete efforts to improve the competences related to these concepts in the nursing college education field or personally are required.

## Figures and Tables

**Table 1:** General characteristics of participants.

Characteristics		Frequency	Rate (%)
Age	<25 years 25–30 31–40 41–50 50<	1 90 145 52 12	0.3 30.0 48.3 17.3 4.0
Gender	Male Female	13 287	4.3 95.7
Marital status	Single Married	160 140	53.3 46.7
Academic record	Junior college University Graduate school	43 209 48	14.3 69.7 16.0
Type of work	3 shifts Full-time Other	214 63 23	71.3 21.0 7.7
Position	General nurse Senior nurse Charge nurse	215 62 23	71.6 20.7 7.7
	Total	300	100

**Table 2:** Technical metrics of the measurement variables (*n* = 300).

Latent Variable	Observed Variable	Mean	Standard Deviation	Skewness	Kurtosis
Psychological well-being	Environmental mastery	3.39	0.35	0.074	0.267
	Self-acceptance	3.22	0.37	−0.037	0.143
	Positive relations with others	2.85	0.43	0.461	0.293
	Autonomy	2.30	0.32	0.326	0.555
	Purpose in life	2.87	0.30	0.314	0.554
	Personal growth	3.04	0.31	0.206	0.039
Emotional intelligence	Self-emotional appraisal	3.89	0.54	−0.618	1.380
	Other’s emotional appraisal	3.71	0.61	−0.487	0.343
	Regulation of emotion	3.52	0.60	−0.143	−0.174
	Use of emotion	3.14	0.70	−0.400	−0.188
Willpower	Limited resource theory	3.81	0.62	−0.204	0.043
	Nonlimited resource theory	3.38	0.74	−0.179	−0.260
Job-efficacy	Job-efficacy	3.60	0.61	−0.359	0.301

**Table 3:** Correlations between the observed variables.

Division	1	2	3	4	5	6	7	8	9	10	11	12	13	14
Psychological Well-being														
Self-acceptance	1													
Environmental mastery	0.50 **	1												
Positive relations with others	0.33 **	0.46 **	1											
Autonomy	0.23 **	0.32 **	0.18 **	1										
Purpose in life	0.41 **	0.69 **	0.51 **	0.27 **	1									
Personal growth	0.36 **	0.49 **	0.53 **	0.21 **	0.60 **	1								
Emotional Intelligence														
Regulation of emotion	0.30 **	0.56 **	0.37 **	0.28 **	0.62 **	0.47 **	1							
Other’s emotional appraisal	0.11 *	0.11	0.24 **	0.01	0.17 **	0.15 **	0.30 **	1						
Use of Emotion	0.30 **	0.34 **	0.16 **	0.19 **	0.28 **	0.20 **	0.40 **	0.14 *	1					
Self-emotional appraisal	0.29 **	0.40 **	0.38 **	0.15 **	0.34 **	0.36 **	0.39 **	0.45 **	0.25 **	1				
Willpower														
Limited resource theory	0.12 *	0.21 **	0.00	0.14 *	0.09	0.11 *	0.21 **	0.00	0.29 **	0.07	1			
Nonlimited resource theory	0.07	0.16 **	−0.06	0.20 **	0.07	0.02	0.19 **	0.01	0.33 **	0.05	0.70 **	1		
Job-Efficacy														
Job-efficacy 1–5	0.38 **	0.44 **	0.32 **	0.24 **	0.47 **	0.40 **	0.58 **	0.28 **	0.26 **	0.35 **	0.21 **	0.13 *	1	
Job-efficacy 6–10	0.23 **	0.42 **	0.30 **	0.24 **	0.40 **	0.30 **	0.30 **	0.01	0.19 **	0.18 **	0.10	0.07	0.44 **	1

Note: * *p* < 0.05, ** *p* < 0.01.

**Table 4:** Fitness index of the measurement model.

Division	Criterion	Fit Measures	Interpretation
Absolute fit index			
GFI	≥0.90	0.94	Fitness
AGFI	≥0.85	0.89	Fitness
S-RMR	≤0.08	0.04	Fitness
Incremental fit measures			
NFI	≥0.90	0.91	Fitness
IFI	≥0.90	0.87	Good
TLI	≥0.90	0.91	Fitness
CFI	≥0.90	0.94	Fitness
Parsimonious fit measures			
PRATIO (Parsimony Ratio)	≥0.50	0.70	Fitness
PNFI	≥0.50	0.63	Fitness
PCFI	≥0.50	0.65	Fitness
Other measure			
RMSEA	≤0.10	0.07	Fitness

Note: GFI: Goodness of Fit Index; AGFI: Adjusted Goodness of Fit Index; S-RMR: Standardized-Root Mean Square Residual; NFI: Normed Fit Index; IFI: Incremental Fit Index; TLI: Tucker Lewis index; CFI: Comparative Fit Index; PNFI: Parsimony Normed Fit Index; PCFI: Parsimony Comparative Fit Index; RMSEA: Root Mean Square Error of Approximation.

**Table 5:** Confirmatory factor analysis of the measurement model.

Variables		Standardized Regression Weight (*B*)	Standard Error	*p*	C.R.
Psychological well-being	Self-acceptance	0.59			
	Positive relations with others	0.58	0.079	<0.001	8.54 ***
	Autonomy	0.44	0.049	<0.001	6.84 ***
	Environmental mastery	0.86	0.071	<0.001	11.04 ***
	Purpose in life	0.83	0.080	<0.001	10.84 ***
	Personal growth	0.66	0.061	<0.001	9.34 ***
Emotional intelligence	Regulation of emotion	0.43			
	Other’s emotional appraisal	0.40	0.08	<0.001	5.60 ***
	Use of emotion	0.90	0.09	<0.001	6.54 ***
	Self-emotional appraisal	0.41	0.77	<0.001	6.75 ***
Willpower	Limited resource theory	0.87			
	Nonlimited resource theory	0.88	0.17	<0.001	5.94 ***
Job-efficacy	Job-efficacy 1–5	0.77			
	Job-efficacy 6–10	0.52	0.12	<0.001	7.24 ***

Note: *** *p* < 0.001.

**Table 6:** Relationships between the human effects of the measurement model.

Directions	Standardized Regression Weight (*B*)	Standard Error	*p*	C.R.
Psychological well-being →emotional intelligence	0.71	0.86	<0.001	9.10 ***
Psychological well-being →willpower	0.27	0.10	<0.001	3.89 ***
Psychological well-being →job-efficacy	0.53	0.12	<0.001	4.27 ***
Emotional intelligence →job-efficacy	0.31	0.11	<0.01	2.85 **
Willpower →job-efficacy	0.03	0.05	0.067	0.53

Note: ** *p* < 0.01, *** *p* < 0.001.

**Table 7:** Mediated effect analysis.

Directions					Direct Effects	Indirect Effects	Gross Effects
Psychological well-being	→			Emotional intelligence	0.71 ***	-	0.71 ***
Emotional intelligence	→			Job-efficacy	0.31 **	-	0.31 **
Psychological well-being	→	Emotional intelligence	→	Job-efficacy	0.53 ***	0.23 **	0.76 ***

Note: ** *p* < 0.01, *** *p* < 0.001.

## Data Availability

The data are not publicly available due to ethical reason.
